# Delirium in a Relatively Young Person due to COVID-19 Infection

**DOI:** 10.1155/2023/6215386

**Published:** 2023-03-23

**Authors:** Soumitra Das, Pablo Melgar, Aniqa Abdul Rasool, Adarsha Adhikari, Rudresh Patel, Lorenzo Abednego B. Adre, Marylaine J. Lopez

**Affiliations:** ^1^Royal Melbourne Hospital, Australia; ^2^Aga Khan University, Karachi, Pakistan; ^3^Gandaki Medical College, Pokhara, Nepal; ^4^Nova Southeastern University Kiran C. Patel College of Osteopathic Medicine, Florida, USA; ^5^Manila Doctors Hospital, Philippines

## Abstract

The coronavirus disease (COVID-19) pandemic, caused by the severe acute respiratory syndrome-coronavirus-2 (SARS-CoV-2 virus), has significantly impacted global health. It can present a range of complications, from asymptomatic to severe respiratory distress syndrome. It has also been linked to complications in multiple organ systems, including neurological symptoms such as headaches and encephalopathy. Delirium, characterized by acute confusion, is common in older adults and associated with prolonged hospital stays and elevated mortality rates. We present a case study of a young mother with a prior medical history of mild to moderate depression who experienced an episode of delirium consequent to a COVID-19 infection. The initial manifestation of her illness was mild diarrhea, but as her condition worsened, she began exhibiting symptoms of delirium. These symptoms include confusion, agitation, sleep disturbance, and disordered behavior. The delirious episode was brief and effectively managed with small doses of psychotropic medications to control aggressive behavior. Upon resolution, no additional treatment was deemed necessary. This case underscores the wide-ranging effects of COVID-19 on physical and psychological well-being and highlights the importance of considering symptoms beyond those associated with respiratory distress.

## 1. Introduction

The coronavirus disease 2019 (COVID-19) caused by SARS-CoV-2, a member of the coronavirus family, was first declared as a pandemic in March 2020 by the World Health Organization [1, 2]. Since then, the disease has led to the loss of life and challenged health systems around the world. There were an estimated 7.5 million hospitalizations and 921 thousand mortalities due to COVID-19 in the United States alone [3]. The European Burden of Disease Network developed a consensus methodology estimating the impact of morbidity and mortality in terms of years lived with disability (YLD) and years lost to premature death (YLL), respectively [4], urging a need for a combined approach that involves epidemiology, surveillance, accurate diagnosis, and prophylaxis to address the global pandemic [2].

The clinical manifestations of COVID-19 vary, ranging from being an asymptomatic carrier and mild respiratory disease to acute respiratory distress syndrome (ARDS) and chronic fatigue syndrome [5, 6]. COVID-19 affects multiple organ systems apart from the respiratory system. Previous studies have shown that the disease may also have hematologic, cardiovascular, renal, gastrointestinal, endocrinologic, and neurologic manifestations [2, 6–10]. Most infections are said to be asymptomatic or mild [11]. Though severe disease may still occur in any individual, individuals of advanced age or with certain medical conditions are more at risk. Clinical manifestation typically occurs after a median incubation of 6.57 days [12], with initial symptoms of cough, myalgias, and headache, including diarrhea, sore throat, and smell or taste abnormalities. Some patients who initially present with mild symptoms may progress in severity and develop neuropsychiatric complications like stroke, seizure, encephalitis, and delirium [6]. A study done in Chicago showed that some common neurological manifestations such as neurologic myalgias, headaches, encephalopathy, dizziness, dysgeusia, and anosmia were independent risk factors for severe COVID-19 infection (OR 4.02 *P* value < 0.001) and younger age (OR 0.982 *P* value = 0.014) [13]. Patients who presented with any neurologic symptoms were found to be younger than those without (mean age 57.53 vs. 62.98 *P* value 0.005). On the other hand, patients who developed encephalopathy were older than those who did not (OR 1.06 *P* value < 0.001) [13].

Delirium is an acute confusion state that typically affects older adults and is marked by fluctuating levels of consciousness, disorientation, inattention, and other cognitive impairments. The infection can cause cerebral inflammation leading to delirium and long-term functional impairment [14]. It is primarily seen in the older population due to prolonged hospitalization and in those who require intensive care unit (ICU) admissions due to COVID-19. It is linked to negative outcomes like an increased length of hospital stay and mortality [15]. However, the presentation of delirium is more common in the older population, even with COVID-19 infection [15].

Although COVID-19-related delirium is a frequent manifestation, there is still a lack of information and reporting in the literature about this manifestation in younger individuals. We aimed to report an interesting case of a relatively young individual who developed delirium, which lasted for a short period without any changes in her biological parameters.

## 2. Case Presentation

X is a 39-year-old female, mother of 2 children with full-time work. X presented to a general practitioner with mild diarrhea, which she initially attributed to a fast-food meal. On the following day, as her diarrhea subsided, she decided to go out shopping, but once at the shops, X thought she and her daughter were in danger, did not feel safe, and decided to return home. When she arrived home, she dropped her phone in the toilet as X believed she was being monitored. Shortly after using the landline, X spoke with her younger sister, who noticed that she had a significant degree of incoherent speech, appeared confused, spoke about bizarre topics, and said things out of character, like making cocaine from sugar. The next day, she was expressing bizarre, sexualized ideas to her sister. Then, X went to the street and banged on car windows, so her sister immediately called emergency services. X was escorted to the emergency department (ED) for further care. At the ED, she was mechanically restrained because she was agitated, disorganized, and threatened to leave the ED. The patient at the time of admission was disoriented as to time and place and was oriented to her sister. She initially calmed down on the persuasion of her sister, however, quickly became verbally aggressive towards the physician and her sister, which according to the informant has rarely been used by the patient. The patient was a housewife with a nonsignificant medical history and a past psychiatric history of moderate depression in remission. She received Haldol 10 mg intramuscular (IM). She became COVID-19 positive on polymerase chain reaction (PCR) tests on the first day of the ED presentation, as mandatory testing in any ED in Victoria. Her chest X-ray, computed tomography (CT) scan of the brain, electrolytes, complete blood count, liver function tests (LFTs), and thyroid profile were all within the normal range. On the fourth day, symptoms were significantly improved, with no more paranoid thoughts or hallucinations. X developed insight into the recent events and was acknowledged to have been unwell. X was diagnosed with delirium due to significant confusion, extreme agitation needing mechanical restraint, sleep impairment, and disorganized behavior. The episode was short lasting (4 days) without needing further psychotropic treatment. At the end of the fourth day, there was a complete resolution of symptoms aligned with the severe infective phase of COVID-19 in the first seven days. X has a history of mild to moderate depression, diagnosed and managed by her general practitioner. She never needed any contact with public mental health or a psychiatrist.

## 3. Discussion

In our case, patient X presented with gastrointestinal (GI) symptoms leading to delirium. Generally, GI and respiratory symptoms are common triggers before the acute confusion state [16]. The SARS-CoV-2 virus entry in the host cells is mediated through the angiotensin-converting enzyme-2 (ACE-2) receptor. The lung and GI tract are the principal sites of ACE-2 receptor expression in the body. However, endothelial cells of the brain also express the ACE-2 receptor protein, which may mediate the entry of the SARS-CoV-2 virus into the CNS, leading to psychological manifestations of COVID-19 [6, 17]. Hyperactive delirium is a common presentation in COVID-19 infection, as in our case. Generally, the presentation is mixed with language disorder, disorganized behavior, and perceptual disturbances [18]. Significant sexualized behavior in a COVID-19-related delirium is rarely reported. Mawhinney et al. reported one such case of neurotropism of COVID-19 infection where a young patient presented with an acute manic episode and confession of past homosexual encounters and other bizarre sexual behaviors as described by his wife [19]. In our case, patient X also had bizarre sexualized ideas that she reported to her sister.

In our case, the possible mechanism behind delirium could be a direct central nervous system (CNS) invasion of COVID-19 or an inflammatory reaction due to systemic infection, as other environmental-related conditions are minimal due to the shorter hospital stay [20]. To manage patient X, a small dose of psychotropics for two days was sufficient to control aggression; however, mechanical restraint was necessary to ensure the safety of patient X and others in the medical ward. As per the previous findings in the literature, preexisting mood disorders are common, which is present in our case as well [14]. However, as the episode was a decade ago without much functional impairment, no information was available. The CT brain scan did not show any preexisting conditions. X was followed for a month after her discharge in a community mental health setting. She showed no signs of relapse from mood/psychotic symptoms or delirious behavior. She was successfully discharged from the community mental health services following one month of stability in her mental health.

COVID-19 delirium is a frequent psychiatric manifestation, yet data related to COVID-19 delirium in younger individuals is scarce, with only a handful of studies reporting delirium in young patients, and a major chunk of literature has assessed delirium in older individuals. Hospitalized COVID-19 individuals' total incidence of delirium, compared to that of hospitalized non-COVID-19 patients, varied from 6% to 56%. Delirium was linked to negative outcomes like prolonged hospital stay and death [20]. A comparative study by Sâbru et al. compared COVID-19-related psychiatric manifestations in older vs. younger patients. It indicated that younger patients with COVID-19 frequently presented with delirium, bizarre behavior, and hallucinations compared to older adults. They also showed that these young adults are more likely to be diagnosed and fully recover; the onset is often abrupt, may last for weeks or months, or maybe as short-lived as a few hours to days [21].

As this is a rare presentation in a younger individual, the case might be a critical addition to the existing literature on eliciting COVID-19 infection-related delirium.

## 4. Conclusion

COVID-19 is a novel disease that has effects on multiple organ systems. One of the documented clinical presentations of COVID-19 is delirium. The patient in our case is a 39-year-old female with a previous history of clinical depression who initially presented with agitation, incoherent speech, and hallucinations. She was admitted to a hospital, and diagnostic studies were done, with the COVID-19 PCR test being the only positive result. She improved after four days of admission with no recurrence of incoherent speech or hallucinations. A previous study has shown that patients who present with delirium are older compared to COVID-19 who did not present with delirium. This same study also showed that neurological symptoms are an independent risk factor for illness severity. Since the patient in our case is a relatively young female with a previous history of psychiatric illness, this case could be a critical addition to the literature on delirium in COVID-19.

## 5. Limitations

More research is required for the enhancement of psychiatric awareness among the population regarding pandemics like COVID-19. One of the limitations of this case is that a very long follow-up of the case was not done even though the patient had a history of prior psychiatric illness. Additionally, further inquiry and follow-up regarding bizarre sexual ideas in our patient to rule out other causes were not carried out by us.
